# Mechanism of Action and Therapeutic Potential of the β-Hairpin Antimicrobial Peptide Capitellacin from the Marine Polychaeta *Capitella teleta*

**DOI:** 10.3390/md20030167

**Published:** 2022-02-25

**Authors:** Victoria N. Safronova, Pavel V. Panteleev, Stanislav V. Sukhanov, Ilia Y. Toropygin, Ilia A. Bolosov, Tatiana V. Ovchinnikova

**Affiliations:** 1 M.M. Shemyakin & Yu.A. Ovchinnikov Institute of Bioorganic Chemistry, The Russian Academy of Sciences, Miklukho-Maklaya Street, 16/10, 117997 Moscow, Russia; victoria.saf@ibch.ru (V.N.S.); ibch@inbox.ru (P.V.P.); ssv@ibch.ru (S.V.S.); bolosov@ibch.ru (I.A.B.); 2 V.N. Orekhovich Research Institute of Biomedical Chemistry, 119121 Moscow, Russia; toropygin@rambler.ru

**Keywords:** antimicrobial peptide, capitellacin, site-directed mutagenesis, antimicrobial resistance, antibiofilm activity

## Abstract

Among the most potent and proteolytically resistant antimicrobial peptides (AMPs) of animal origin are molecules forming a β-hairpin structure stabilized by disulfide bonds. In this study, we investigated the mechanism of action and therapeutic potential of the β-hairpin AMP from the marine polychaeta *Capitella teleta*, named capitellacin. The peptide exhibits a low cytotoxicity toward mammalian cells and a pronounced activity against a wide range of bacterial pathogens including multi-resistant bacteria, but the mechanism of its antibacterial action is still obscure. In view of this, we obtained analogs of capitellacin and tachyplesin-inspired chimeric variants to identify amino acid residues important for biological activities. A low hydrophobicity of the β-turn region in capitellacin determines its modest membranotropic activity and slow membrane permeabilization. Electrochemical measurements in planar lipid bilayers mimicking the *E. coli* membrane were consistent with the detergent-like mechanism of action rather than with binding to a specific molecular target in the cell. The peptide did not induce bacterial resistance after a 21-day selection experiment, which also pointed at a membranotropic mechanism of action. We also found that capitellacin can both prevent *E. coli* biofilm formation and destroy preformed mature biofilms. The marked antibacterial and antibiofilm activity of capitellacin along with its moderate adverse effects on mammalian cells make this peptide a promising scaffold for the development of drugs for the treatment of chronic *E. coli* infections, in particular those caused by the formation of biofilms.

## 1. Introduction

The rapid emergence of resistant bacteria is occurring worldwide, compromising the effectiveness of conventional antibiotics. Therefore, new antimicrobial therapeutics are required to cure chronic infections, in particular those caused by the formation of biofilms. Antimicrobial peptides (AMPs) play a key role in the immune system of invertebrates and vertebrates, as well as in plants, and they are considered as prototypes of new antibiotics [1]. Among all structurally diverse AMPs, β-hairpin peptides attract particular attention. Their main advantages include a broad-spectrum activity, rapid bactericidal action, low development of resistance, and increased structural stability to proteolytic degradation. At the same time, they have some serious limitations, such as hemolysis or/and cytotoxicity toward mammalian cells and a decrease or loss of their antibacterial activity in physiological salt conditions [2,3].

Capitellacin from the marine polychaeta *Capitella teleta* overcomes some of these limitations. Its β-hairpin structure was proved by CD and NMR spectroscopy in our previous study [4]. Capitellacin demonstrated a broad-spectrum activity against a wide panel of bacteria including drug-resistant strains, which makes it a promising candidate for further optimization and possible systemic use. In our previous work [4], it has been shown that capitellacin possessed a low amphipathicity and did not dimerize both in aqueous solution and in lipid-mimicking environments. In contrast to other known β-hairpin AMPs, capitellacin was inefficient in the disruption of cytoplasmatic membrane integrity and inhibition of bacterial translation. We assumed that capitellacin kills cells via a non-lytic mechanism and probably interacts with any intracellular bacterial targets. At the same time, the peptide is capable of dodecyl-phosphocholine micelle and PE/PG (2:1) liposome binding [4]. Thus, the mechanism of its antibacterial action is still obscure.

Marked difference are observed between the mechanism of action of capitellacin and tachyplesin-1 despite their high structural similarity. However, the mechanism of action of tachyplesin-1 is based on its multiple cellular effects; therefore, detailed evidence of this process is not yet demonstrated. It has been shown that the primary and main target of tachyplesin-1 is the cell membrane, due to the high affinity of the peptide for LPS, which leads to the destabilization of the outer membrane [5,6,7]. On the other hand, it was believed that tachyplesin-1 was able to span the lipid bilayer, which led to the formation of anion-selective pores followed by translocation (“barrel-stave” model) [8]. Additionally, the leakage of tachyplesin-1 molecules into the cytoplasm and the efflux of potassium ions led to an increased permeability, which could also be a potential mechanism for the destruction of the bacterial membrane [9,10]. In other cases, it has been demonstrated that tachyplesin-1 translocated into the inner leaflet of the bilayer, caused a phospholipid flip-flop, and formed toroidal pores [10]. Several proposed mechanisms of action of tachyplesin-1 are primarily related to the bacterial strain, concentration of the peptide, and time of its exposure. Secondly, distinct mechanisms of action of tachyplesin-1 against Gram-positive and Gram-negative bacteria have been shown. Tachyplesin-1 could translocate across the *E. coli* membrane via pore formation, which led to leakage of intracellular components. In the case of *S. aureus*, the membrane was depolarized but not destroyed [6]. In addition to direct membrane binding, it could also be accumulated within cells and bind to a minor DNA groove or inhibit the intracellular esterases [6,11]. Despite a substantial structural homology between tachyplesin-1 and capitellacin, the basis of the differences in their mechanisms of action remains unknown and is the focus of our attention.

This paper focuses on the exploration of the capitellacin mechanism of action and on the assessment of the contribution of distinct amino acid residues to its biological properties. Here, a set of capitellacin analogs (CT2, CT3) and tachyplesin-inspired chimeric variants (CT4–CT7) were obtained and analyzed. In particular, the antibacterial, cytotoxic, and antibiofilm properties of capitellacin analogs and the ability of capitellacin to permeabilize both living bacterial cells and model systems mimicking bacterial membranes were studied.

## 2. Results and Discussion

### 2.1. Design of Capitellacin Variants and Tachyplesin-Inspired Chimeric Analogs

**Figure 1:**
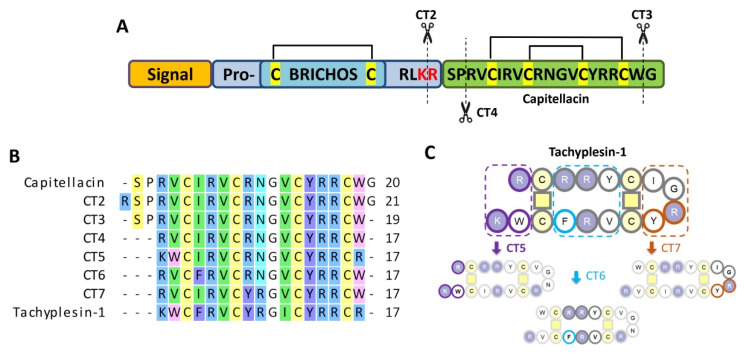
(**A**) Preprocapitellacin structure; (**B**) alignment of capitellacin analogs; (**C**) design of tachyplesin-inspired chimeric analogs. The putative natural processing sites are indicated with scissors.

First, we performed a structure–activity relationship study of capitellacin to improve its antimicrobial activity and identify amino acid residues contributing to the low membranolytic activity and cytotoxicity of the peptide. Since mature capitellacin was not isolated from animal tissues, it was previously decided to obtain its recombinant analog. The main obstacle at this stage was the lack of data on the exact structure of mature natural AMP. A closer look at the amino acid sequence of the precursor protein (procapitellacin) suggests the presence of several alternative processing sites for the mature peptide (Figure 1A). BRICHOS-related AMPs such as arenicins are cleaved from the precursor protein by furin protease at the site after two positively charged amino acid residues (Lys-Lys↓, Lys-Arg↓, or Arg-Arg↓) [12]. Similar processing sites are conventional for hydrolysis by furin protease during the maturation of other invertebrate AMPs [13] as well as most amphibian AMPs [14]. Therefore, we suggested 20-residue *C*-terminal peptide as the most probable variant of a mature capitellacin. At the same time, the processing of another BRICHOS-related peptide, alvinellacin, occurs at an alternative site—the lysine residue in the Lys↓Arg dibasic site [15]. Thus, the *N*-terminal residue of the mature peptide could be both serine (capitellacin) or arginine residue (designated as CT2). Notably, the *C*-terminal residue of procapitellacin is glycine. It is known that the presence of glycine in this position in AMPs from animals, including invertebrates, is often a signal for an additional stage of post-translational modification by the enzyme peptidylglycine alpha-amidating monooxygenase (PAM), which cleaves the *C*-terminal glycine residue, followed by amidation of the carboxyl group of the preceding amino acid residue. Such a modification has been previously described for the antimicrobial peptide hedistin from *Nereis diversicolor* [16], and it is also quite common for many cathelicidins [17]. Therefore, in this work, we also decided to obtain and analyze the biological activity of the 19-residue CT3 analog lacking *C*-terminal glycine. In this study, we also applied site-directed mutagenesis and recombinant production to the bioengineering of the shortened capitellacin-like peptides. Peptide chain length is considered to be of key importance when optimizing an AMP structure [18]. Considering this structural similarity to tachyplesins and the pronounced wide-spectrum antimicrobial activity of these natural molecules, tachyplesin-inspired chimeric peptides were also obtained and analyzed as potential antimicrobial agents. Peptide CT4 was designed as a shortened 17-residue capitellacin variant, while CT5, CT6, and CT7 analogs were engineered by incorporating the functionally important residues from tachyplesin-1 onto CT4. The designed peptides contained, respectively, the terminal (K1R, V2W, W17R), central (I4F), and β-turn (R8Y, N9R) parts of a tachyplesin molecule.

By analogy with our previous study, here we used heterologous expression in the bacterial system of the peptides fused with modified thioredoxin A—a highly soluble carrier protein that was successfully used for the recombinant production of other β-hairpin AMPs [19]. All the fusion proteins were expressed in *E. coli* BL21 (DE3) cells, and the obtained total cell lysates were fractionated by affinity chromatography. After the purification and cleavage of the fusion proteins, reverse-phase high-performance liquid chromatography (RP-HPLC) was used to obtain target AMPs. RP-HPLC was performed using a Reprosil-pur C18-AQ column at a flow rate of 2 mL/min in a linear gradient of solution B (80% acetonitrile, 0.1% TFA) in solution A (5% acetonitrile, 0.1% TFA) for 75 min (Supplementary Figure S1). MALDI mass spectrometry analysis of the main fractions showed that the measured monoisotopic *m*/*z* matched well with the calculated molecular masses of the protonated ions [M+H]^+^ of the corresponding peptides (Table 1). The fractions with confirmed masses were dried in vacuum and repurified using the analytical column (Symmetry 300 C_18_) at a flow rate of 1 mL/min in a linear gradient of solution B in solution A: 0–100% for 50 min. All peptides demonstrated a purity greater than 95% (Supplementary Figure S2). The final yields of the peptides were 4.6–7.2 mg per 1 L of the culture.

### 2.2. Antimicrobial Activity of Capitellacin Analogs

The antibacterial activity of the obtained peptides was determined by two-fold serial dilutions in 96-well plates against a panel of opportunistic Gram-positive and Gram-negative bacteria. Serial dilutions were made in 0.1% BSA (bovine serum albumin) to minimize the absorption of peptides on the surface of the plate wells. We also used Mueller–Hinton medium (MH medium) supplemented with 0.9% NaCl, which might constrain an absorption of the peptides to the bacterial surface and therefore decrease the activity of many cationic AMPs [20]. All capitellacin variants exhibited comparable in vitro antimicrobial activity, with the highest potency observed against Gram-negative bacteria (Table 2). The greatest antimicrobial potential among all analogs was shown by CT2 against Gram-negative bacteria (GM value of 0.46). Therefore, the extra *N*-terminal arginine residue facilitates the selectivity of capitellacin against bacterial pathogens. No substantial effect of the structure in different processing variants (CT2, CT3) on the biological activity was detected. We assumed that the previously used 20-residue peptide reflected the properties of the proposed natural variant, whatever the processing site. In most cases, shortened capitellacin analogs showed the same activity level as a full-length peptide, while CT5 demonstrated a slight decrease in activity that was likely related to its lower hydrophobicity as compared to all analogs, which correlated with the retention time on RP-HPLC (Table 1). Notably, only CT7 chimeric variant demonstrated activity similar to tachyplesin-1 against Gram-positive bacteria (GM of 2 vs. 1.59), but not Gram-negative bacteria (GM of 0.55 vs. 0.15).

### 2.3. The Structure of β-Turn Region Defines Low Membranotropic Activity of Capitellacin

**Figure 2:**
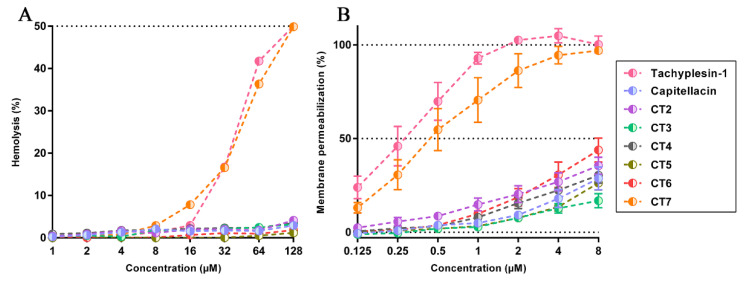
(**A**) Hemolytic effect on human red blood cells after 1.5 h exposure of capitellacins and tachyplesin-1; (**B**) the effect of the peptides at different concentrations on the permeability of the cytoplasmic membrane of *E. coli* ML-35p after 1.5 h (ONPG assay). Data are the mean ± SD of three independent experiments.

To examine the ability of the peptides to permeabilize cytoplasmic membranes of bacterial and mammalian cells, the ONPG test and hemolysis assay were used, respectively. Previously, it was shown that capitellacin is significantly less toxic to both red blood cells and cell lines of human embryonic fibroblasts (HEF) in contrast to tachyplesin-1. The hemolytic activity of AMPs against a suspension of human erythrocytes was determined by the method of double serial dilution in buffered saline followed by incubation for 1.5 h (Figure 2A). It was shown that the maximum level of lysis for each peptide was no more than 3–4% at a concentration of 128 μM, with the exception of CT7, for which this value was almost 50%. Notably, a similar HC50 value of 128 μM was observed for tachyplesin-1. Next, we used ONPG assay to estimate *E. coli* inner membrane permeabilization by the peptides. Again, most of the analogs exhibited only a modest membranolytic effect at MIC (0.25–0.5 µM) after 1.5 h of incubation, while CT7 and tachyplesin-1 had a similar pronounced effect in the same conditions (Figure 2B). Thus, amino acid substitutions in the β-turn (R8Y, N9R) part of capitellacin are likely to cause a significant change in the mechanism of action. Interestingly, a point substitution which lowers the hydrophobicity of tachyplesin-1 in the β-turn region, in particular, (Y8S) or (I11S), decreases its ability to permeabilize bacterial cells as well as to cause cytotoxic effects against mammalian cells [21,22]. Here, we can conclude that the fairly hydrophilic structure of the β-turn region defines the low membranotropic activity of capitellacin in comparison with tachyplesin-1.

### 2.4. Capitellacin Does Not Induce Bacterial Resistance

**Figure 3:**
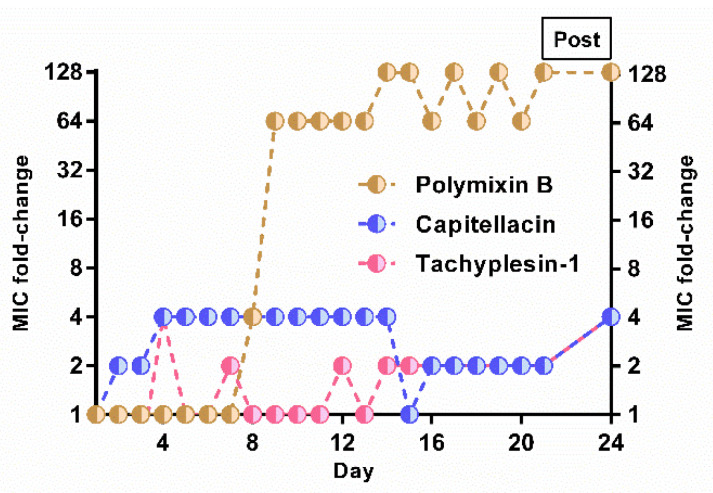
Induction of *E. coli* MDR CI 1057 resistance to capitellacin, tachyplesin-1, and polymyxin B in Mueller–Hinton medium containing added NaCl. Initial MIC values were: capitellacin 1 (0.5 µM), tachyplesin-1 (0.125 µM), and polymyxin B (0.125 µM). Bacteria that grew at the highest concentration of AMPs on the final passage (day 21) were passaged a further 3 times on drug-free agar plates before determining the final MIC value (“Post”).

It is believed that there is little or no development of resistance without significant fitness costs to cationic membrane-targeted AMPs, since it requires crucial changes in the structure and electrophysiological properties of the cell membrane. Therefore, at the next stage we attempted to select capitellacin-resistant *E. coli* strains using the serial passage method which allows the registering of MIC values after each transfer [23]. Tachyplesin-1 and polymyxin B were taken as reference drugs (Figure 3). To assess the stability of the induced mutations obtained after 21 days of passages, an additional 3 days of passages was carried out in the absence of any drug. The rate of stable resistance to both capitellacin and tachyplesin-1 was quite low (4-fold increase in MIC) compared with the lipopeptide antibiotic polymyxin B (128× MIC). The resistance to capitellacin was registered much earlier, resulting in 4× MIC after just 4 passages. Notably, 64-fold increases in MIC value were registered after just 9 passages in the bacterial strain MDR CI 1057 subjected to selection by polymyxin B. The induction of resistance to capitellacin against *E. coli* BW25113 was also investigated, and a similar 2–4-fold increase in MIC (initial MIC of 0.5 μM) was detected after a similar 3-week experiment performed in duplicate (graphical data not shown). While the ability of capitellacin to bind any specific molecular targets on bacterial membranes or inside the cell was not excluded, it demonstrated a low tendency to induce stable resistance in vitro, which pointed to a membranotropic mechanism of action. A similar level of resistance growth was previously shown for a number of membrane-targeted AMPs, which may indicate the absence of specific targets for capitellacin as well [24,25]. In this case, a small increase in resistance may be associated with any changes in the structure of the membrane.

### 2.5. A Study of Capitellacin-Induced Conductance of Planar Lipid Membranes

**Figure 4:**
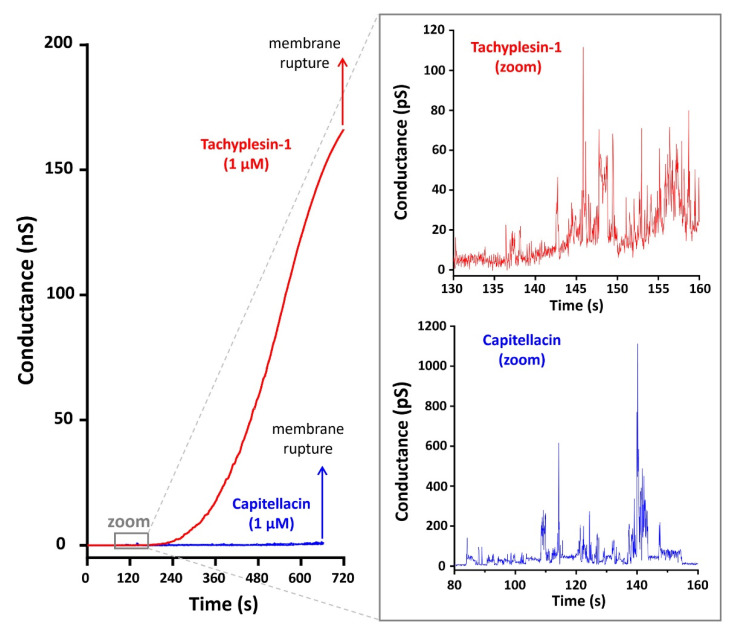
The peptide-induced changes in conductance after an addition of tachyplesin-1 and capitellacin. Zoomed-in records of single fluctuations of conductance induced by the peptides are presented in the right panel. Each peptide was added to both sides of BLM in the experiment. The applied voltage was 50 mV and the buffer solution contained 0.154 M NaCl and 5 mM HEPES (pH 7.4).

The Mueller planar lipid bilayer technique mimicking the plasma membrane of Gram-negative bacteria was used for the detailed study of peptide-induced membrane permeabilization by capitellacin and tachyplesin-1. Figure 4 shows currents through planar bilayer lipid membranes (BLM) formed using the phospholipid mixture consisting of ∼70% zwitterionic PE and ∼30% anionic PG and DPG (weight percent). The data obtained demonstrate that both peptides at a concentration of 1 µM induced conductivity fluctuations within a few minutes after addition to the membrane. At 140 s, the frequency of fluctuations increased for tachyplesin-1 and, as a result, the integral conductivity of the membrane increased to a maximum value of ~180 nS (Figure 4). This indicates the formation of many conducting pores, which ultimately led to the destabilization and rupture of the membrane 600–720 s after the addition of the peptide. In contrast, the addition of capitellacin resulted in only a slight increase in the integral conductivity to 2 nS followed by membrane destruction. The observed conductance revealed the formation of heterogeneous populations of ion-permeable pores having a relatively low conductivity in the range of 5–50 pS and 20–170 pS for tachyplesin-1 and capitellacin, respectively. A statistical analysis of pore conductivity was not performed due to the small number of observed events. It should also be noted that capitellacin caused short-term, large-amplitude bursts of conductivity reaching 1 nS or more. The membrane conductivity induced by tachyplesin-1 was characterized by the absence of high-conductivity pores. With maximum single fluctuations of 100 pS, the conductivity increased quite smoothly and tended to reach a plateau. Assuming the cylindrical geometry of the conducting pore filled with electrolyte, its effective diameter was also calculated: ~5.1 Å for a conductivity level of 50 pS, which is in good agreement with the calculated diameter of the conducting pore formed by known β-hairpin peptide arenicins in a membrane of the same lipid composition [26]. At the same time, the change in conductivity caused by tachyplesin-1 occured in a shorter time and in a much wider range as compared with arenicins. 

Thus, on the basis of the experimental data obtained, the following assumptions can be made. Capitellacin predominantly accumulates on the membrane surface and, in general, does not integrate into the bilayer to form stable pores. Such an increase in the surface concentration of the peptide leads to a weakening of the interaction within the bilayer, the deformation of the bilayer, and the appearance of pores with high conductivity, which ultimately leads to the destruction of the membrane. To the best of our knowledge, this study is also the first report of tachyplesin-induced BLM permeabilization. In contrast to capitellacin, tachyplesin-1 is able to pass from the surface to the hydrophobic region of the membrane, with the formation of conducting pores, thereby not significantly affecting the stability of the membrane. Most likely, toroidal pores with different conductance are formed, which is in agreement with the previous studies of tachyplesin-1 [10].

### 2.6. Capitellacin Demonstrates a Delayed Cell Membrane Permeability Effect as Compared to Tachyplesin-1

**Figure 5:**
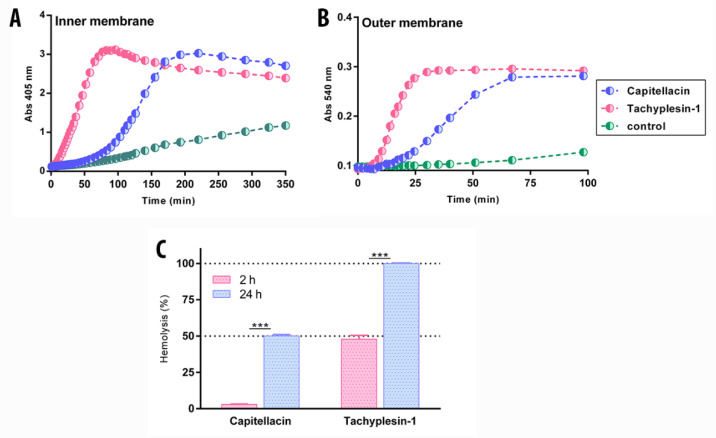
Evaluation of the increase in membrane permeability of *E. coli* ML-35p caused by peptides at a concentration of 1 µM using chromogenic markers: (**A**) the products of o-nitrophenyl-D-galactoside (ONPG, abs 405 nm) hydrolysis; (**B**) nitrocefin, abs 540 nm. Control measurements were carried out without peptides and showed no penetration of substrates through bacterial membranes. The experiment was carried out twice; the curve behaviors were similar. (**C**) Comparison of hemolytic activity of capitellacin and tachyplesin-1 at a concentration 128 µM against erythrocytes from human after 2 h and 24 h incubation (hemoglobin release assay). ±SD of at least two independent experiments, *** *p* ≤ 0.001.

In our previous studies, β-hairpin AMPs arenicin-1, protegrin-1, and tachyplesin-1 caused almost full membrane permeabilization after 60 min at a concentration several-fold higher than MIC [4,19,27]. Notably, all the peptides appeared to act via a toroidal pore mechanism against live cells. Moreover, both arenicin-1 and protegrin-1 formed noncovalent dimers in a membrane-mimicking environment. In contrast, capitellacin, even at the concentration of 32× MIC, demonstrated only a partial effect of cell membrane permeabilization after the same time period [4]. At the same time, new BLM data and our previous structural data in a membrane-mimicking environment [4] point to the carpet mechanism of action. Therefore, capitellacin likely demonstrates slow kinetics of membrane permeability. To validate this hypothesis, we performed membrane permeability tests with longer incubation periods of up to 24 h (Figure 5). *E. coli* ML35p, due to a deficiency of lactose permease and the constitutive synthesis of the β-galactosidase enzyme in the cytoplasm, was chosen as the test strain. The state of the cytoplasmic/outer membrane was evaluated by its permeability to the chromogenic substrate β-galactosidase (o-nitrophenyl-β-D-galactopyranoside, ONPG) and β-lactamase, an enzyme that transports lactose into the cell (nitrocefin), respectively [4]. As expected, capitellacin was indeed capable of disrupting the integrity of biological membranes in living cells, but demonstrated a delayed membrane permeability effect as compared to other known β-hairpin AMPs. In particular, capitellacin caused the full permeabilization of the inner and outer membrane of *E. coli* after 200 and 70 min, respectively (Figure 5A,B). These data corresponded well with the results of electrophysiological studies on BLM: capitellacin accumulates on the surface layer of the membrane, and when the peptide concentration reaches critical values over time, the lysis occurs. Moreover, capitellacin caused a significant (50%) hemolytic effect after 24 h exposure at 128 µM (Figure 5C). It is significant that the toxic effect of capitellacin after 24 h was observed only at concentrations above 32 µM (64× MIC), which does not limit its potential systemic use as an antimicrobial agent at high therapeutic doses.

### 2.7. Capitellacin Exhibits Anti-Biofilm Activity against E. coli

The overwhelming majority of studies on AMP antibacterial activity are carried out on planktonic cell cultures. However, many pathogenic cells can attach to biotic or abiotic surfaces, and the treatment of such infections associated with biofilm formation is a challenging task for modern medicine. A biofilm constitutes a community of microorganisms embedded in a self-produced protective extracellular polymer matrix consisting of exopolysaccharides, proteins, nucleic acids, and other extracellular inclusions. This barrier prevents the penetration of antibacterial agents into biofilms and allows bacteria to possess a high mutation rate and to exchange resistance genes internally, which leads to drug resistance [28,29]. *E. coli* biofilms are the most common cause of intestinal infections (Crohn’s disease, bleeding), infections of the urogenital tract, catheter-associated infections (prostheses, shunts, intravascular catheters), and medical-device-associated infections [30,31]. In addition to the high antimicrobial activity of AMPs against planktonic cultures and their immunomodulation properties, they can also be potential agents for the treatment of chronic diseases caused by biofilm formation [32,33].

**Figure 6:**
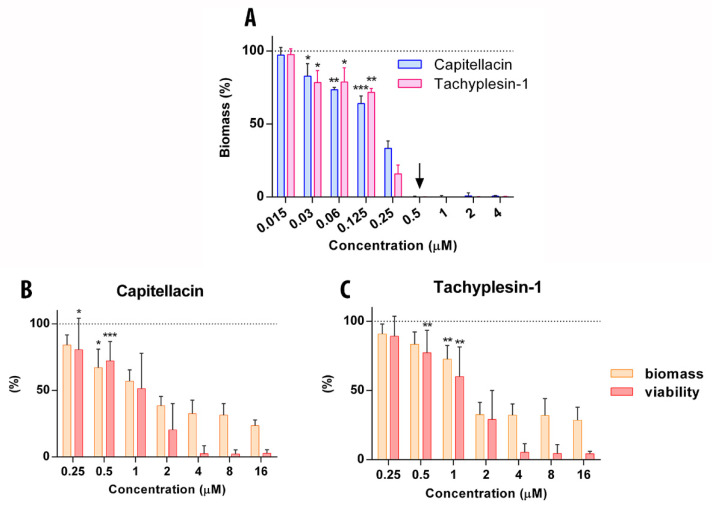
Anti-biofilm activity of capitellacin and tachyplesin-1 against *E. coli* SBS 1936. (**A**) Biofilm formation was established using the crystal violet stain technique. Sterile water was used as solvent for serial dilutions of peptides. Up arrow is MIC of peptides. The ability of capitellacin (**B**) and tachyplesin-1 (**C**) to impact on preformed biofilms of *E. coli* SBS 1936 after overnight exposure. Evaluation of mass of biofilm was assessed using the crystal violet stain technique. The change in the percentage of surviving bacteria in the biofilm was controlled using MTT assay (statistically significant differences vs. the control: * *p* ≤ 0.05, ** *p* ≤ 0.01, *** *p* ≤ 0.001).

At the first stage, different strains of *E. coli* were screened for their ability to form a biofilm on the surface of the plate wells in rich LB medium using the crystal violet staining method. Based on the received data, the *E. coli* SBS 1936 [34] strain demonstrates the highest ability to form biofilms and therefore was selected as a model object (OD_570_ 0.37 ± 0.03). The antibiofilm activity of capitellacin and tachyplesin-1 against the planktonic culture of *E. coli* SBS 1936 in stationary phase was evaluated by the method of double serial dilutions in liquid LB medium. Amphiphilic peptides can be adsorbed on the plate surface; therefore, sterile water and BSA were used as a solvent for the double serial dilutions (it was shown that BSA does not affect either biofilm mass or AMP activity). As shown in Figure 6A, capitellacin and tachyplesin-1 at a concentration of 0.5 μM completely inhibited the formation of *E. coli* biofilms in a dose-dependent manner, which indicated the high efficiency of their antibacterial action.

We assume that the inhibition of biofilm formation may be associated primarily with the bactericidal action of peptides against *E. coli*. It is important that at subinhibitory concentrations these peptides do not stimulate *E. coli* biofilm formation, as was shown under the influence of certain antibiotics on *P. aeruginosa*, such as aminoglycosides and tetracyclines [35,36]. Besides the inhibition of *E. coli* biofilms, a similar effect of reducing the mass of biofilms was observed with the action of capitellacin against a biofilm of *P. aeruginosa*, *K. pneumonia,* and *A. baimannii* (Supplementary Figure S3).

The disruption of mature biofilms (established biofilms) is a complex problem, in particular due to the presence of subpopulations of stationary and persistent cells that possess a reduced metabolic rate and are able to survive in biofilms at high antibiotic concentrations. Therefore, at the next stage, we assessed the ability of peptides to destroy preformed biofilms at 32× MIC within 16 h of treatment (Figure 6B,C). The application of high concentrations of peptides, starting from 8× MIC, ensured a minimum residual viability of bacteria (about 5%), which was determined by a metabolic dye reduction assay using MTT. However, even at lower concentrations, both peptides demonstrated a significant decrease in both the amount of biofilm biomass and the viable bacteria within. Notwithstanding any differences in the mechanisms of action, these peptides demonstrated almost identical antibiofilm effects, due rather to similarities in their physicochemical properties. Nevertheless, even near the MIC value, capitellacin possesses a higher activity against bacteria in biofilms, as well as a higher degree of degradation of the extracellular matrix. Thus, capitellacin is capable of acting on both emerging and pre-formed biofilms and, taking into account its low cytotoxicity, can be considered as a potential agent in further drug development to treat *E. coli*-formed biofilms.

## 3. Materials and Methods

### 3.1. Expression and Purification of the Antimicrobial Peptides

Recombinant AMPs were obtained with the use of *E. coli* BL21 (DE3) heterologous expression system, which is based on a fusion protein that included an 8× His-Tag, carrier protein, and a methionine residue, as described previously [19]. Strain-producers of AMP were received through *E. coli* BL21 (DE3), transformed with the corresponding plasmids. Inoculums were grown in LB (10 g/L peptone, 5 g/L yeast extract, 10 g/L NaCl) medium containing 20 mM glucose, 100 μg/mL ampicillin, 1 mM MgSO_4_, 0,1 mM CaCl_2_, and 0,01 mM FeCl_3_ at 37 °C to the final density of 1.0; next, 0.2 mM isopropyl β-D-1-thiogalactopyranoside (IPTG) was added to the culture of cells. The culture was cultivated at 32 °C for 5 h under rotation at 250 rpm. The cells were grown up to OD_600_ 1.0 and then were induced with 0.2 mM IPTG. The induction was performed at 30 °C for 16 h with a shaking speed of 220 rpm.

Purification of the peptides involved immobilized-metal affinity chromatography (IMAC) of cell lysate, CNBr cleavage of the fusion protein, and RP-HPLC. Further, the cells were centrifuged and sonicated in the 100 mM phosphate buffer containing 6 M guanidine hydrochloride and 20 mM imidazole. The lysed cells were centrifuged at 25,000× *g* for 30 min. Ni-NTA agarose (Qiagen) was used for the purification of the fusion protein under denaturing conditions. Concentrated HCl and an excess of cyanogen bromide was added to the produced fraction to bring it to pH 1.0, then the reaction mixture was allowed to stand at 25 °C for 18 h in the dark. The lyophilized products of the cleavage reaction were dissolved in water. Reserved-phase high-performance liquid chromatography (RP-HPLC) was performed for the final stage of purification of peptides. Further, the required fractions were lyophilized and dissolved in water. The recombinant peptides were characterized by Tricine-SDS-PAGE, MALDI-MS (Bruker Daltonics), and antimicrobial activity testing.

### 3.2. Antimicrobial Assays

The antimicrobial activity of investigated peptides was determined by the method of two-fold serial dilutions in the sterile 96-well flat-bottom polystyrene microplates (Eppendorf #0030730011, Hamburg, Germany). Mueller–Hinton broth was used as a culture fluid. The bacterial strains were cultured in the MH medium at 37 °C for 16 h, then aliquots of culture were diluted with fresh MH and grown to optical density (OD_600_) of 1. The bacterial cultures were prepared in the NaCl-enriched MH broth (42 g/L Mueller–Hinton broth, 18 g/L NaCl) to the final concentration of 1 × 10^6^ colony-forming units (CFU)/mL. In the next step, 50 μL of obtained cell culture was added to 50 μL of peptides to reach the final cell density of 5 × 10^5^ CFU/mL; the compounds had been previously diluted with sterilized 0.1% BSA. After adding the suspension of cells, the plate was incubated at 37 °C for 24 h and 1000 rpm on the plate thermo-shaker ((Biosan, Riga, Latvia). The values of MIC (minimum inhibitory concentration) were identified as the lowest concentration of peptide at which the growth of the culture had not taken place and defined visually, or 0.1 mg/mL of resazurin was added to each well and incubated at 37 °C for 2 h. The tests were carried out at no fewer than two repetitions of free independent experiments, and the MIC of peptides was calculated as an averaged value of these assays.

### 3.3. Hemolytic Assay

The hemolytic activity of compounds was performed using human red blood cells (hRBS), in accordance with the previously described approach [19]. Briefly, hRBS of a healthy male donor were separated from fresh blood and washed 3 times with ice-cold phosphate-buffered saline (PBS pH 7.4) and centrifuged at 500× *g* for 10 min; additionally, citrate buffer was added to prevent clotting. Further, supernatant was discarded and erythrocyte suspension was prepared in PBS as 8% (*v/v*) solution. The test was carried out in the 96-well plate, taking into account two-fold serial dilutions of studied peptides. The plate was incubated at 37 °C for 120 min and on the mixer at 1000 rpm. Then the plate was centrifuged at 1000× *g* for 15 min for the precipitation of erythrocyte. The amount of hemoglobin release was determined by absorption spectroscopy at 405 nm. Triton X-100 (0.1% *v/v*) was used as a positive control, which underwent a complete lysis of erythrocytes, and PBS working solution was used as a negative control. The percentage hemolysis was calculated by the following formula:

$$\mathrm{Hemolysis}\;(\%)=\frac{{\mathrm{OD}}_{405}\left(\mathrm{compound}\right)-{\;\mathrm{OD}}_{405}\;\left(\mathrm{zero}\;\mathrm{lysis}\right)}{{\mathrm{OD}}_{405}\left(100\;\%\;\mathrm{lysis}\right)-{\;\mathrm{OD}}_{405}\;\left(\mathrm{zero}\;\mathrm{lysis}\right)}\times 100\%$$

The assays were conducted three times. The quantitative data were presented as an average means with standard deviations. 

### 3.4. The Effect of Peptides on the Permeability of the Outer and Cytoplasmatic Membrane of E. coli ML-35p

The state of outer and internal membrane of *E. coli* ML-35p was based on its permeability in the presence of antimicrobial peptides and nitrocefin and o-nitrophenyl-β-D-galactopyranoside (ONPG) in the capacity of a substrate of β-lactamase and β-galactosidase in the periplasmatic or cytoplasmatic space, respectively, as described by [37]. If the antimicrobial peptide disturbs the integrity of the outer or cytoplasmatic membrane, the substrate enters the periplasm or cytoplasm and is cleaved by the enzyme; in the process, the cleavage product becomes colored. The ML-35p strain of *E. coli* was used in stationary growth phase; then, the bacterial suspension was dissolved in phosphate-buffered saline (PBS pH 7.4) to reach the final concentration of 2 × 10^7^ CFU/mL. The final concentration of OPNG was 2.5 mM, or 20 µM nitrocefin and 100 mM NaCl was added. The compounds were placed in a 96-well plate with a nonbinding surface (NBS, Corning #3641, Corning, NY, USA) and incubated at 32 °C for at least 180 min and 400 rpm on the plate thermo-shaker. Further, the optical density of the solutions was measured as λ = 405 nm. Control experiments were performed under the same conditions without the addition of a peptide. The optical absorption of the solution after incubation with 5 µM melittin for 1 h was taken as 100%. The absorbance of control wells containing no peptides was subtracted from the absorbance value of each well. Three independent experiments were performed, and the curve patterns were similar for all three series.

### 3.5. Measurement of Biofilm Activity

The ability of antimicrobial peptides to influence the biofilm formation was assessed by the method of serial dilutions in the nutritional medium, as described previously in the published protocol [38]. Briefly, the studied compounds were prepared by two-fold serial dilutions with sterilized water and sterilized 0.1% BSA in 96-well plates to reach the final volume of 50 μL. Next, the cell cultures were grown in LB medium (10 g/L peptone, 5 g/L yeast extract, 10 g/L NaCl) at 37 °C for 24 h; then, aliquots of culture in the stationary phase were diluted with the same medium and 50 μL of the bacterial suspension was added to peptide solutions in the plate to reach the final concentration of 4 × 10^5^ CFU/mL. The plate was incubated at 32 °C for 24 h under rotation at 120 rpm to enable the formation of a biofilm primarily on the wall of the well. The values of MIC were determined as the lowest concentration of peptides at which there was inhibition of cell growth of the microorganism. Further, the planktonic cells were transferred to the new 96-well plate and the cultures of the cells were measured at 570 nm. After removing planktonic cells, the wells were rinsed with 0.8% NaCl three times and the formed biofilms were stained with 150 μL of 0.1% crystal violet (CV, Sigma) for each well. Then, the plates were incubated at 25 °C for 40 min in the dark and the wells were washed with 0.9% NaCl three times again to clean up the excess dye. Next, 120 μL of 30% acetic acid was added to each well and incubated at 25 °C for 15 min for the extraction of CV from biofilms. Further, the resulting extracts were transferred to new plates and the absorption of solution was determined at 570 nm. Every experiment was carried out in triplicate. GraphPad Prism 6 software was used for the statistical processing. 

### 3.6. The Effect of Peptides on Established Biofilms

Biofilms were grown on a 96-well plate at 32 °C for 24 h under rotation at 120 rpm. The ability of peptides to eradicate mature biofilm of *E. coli* SBS1936 was determined using the dying method by CV solution (0.1%), as described above. Bacterial survival in the biofilms was assessed using the MTT assay after incubation with peptide. Then, wells were rinsed with distilled H_2_O or 0.9% NaCl, treated with an MTT solution (Sigma) in PBS, and incubated for 4 h at 37 °C. Next, the medium was removed. The resulting formazan crystals were dissolved in a mixture of EtOH and DMSO (1:1). The optical density of the solution was measured at 570 nm. Bacterial survival was calculated as percent relative to samples without antimicrobial agent. All data were obtained from at least four independent experiments in three repetitions. 

### 3.7. Resistance Induction of Peptides against Bacteria

The overnight culture of *E. coli* MDR CI 1057 was used as the starting inoculum for which the initial MIC value was determined. Further, an aliquot of the bacterial suspension was taken from the first well containing the subinhibitory concentration of the peptide and was diluted with Mueller–Hinton broth supplemented with NaCl. Then, the resulting suspension was added to a solution of peptides (1 passage per 24 h). After 21 serial repeat passages for each peptide, the concentration of peptides at which bacterial growth was possible was subcultured on an agar plate for three days. Next, the final MIC values were established.

### 3.8. Preparation of Planar Bilayers and Electrochemical Measurements

The BLM was made of polar lipid extract from *E. coli* (Avanti Polar Lipids) consisting of PE, PG, and DPG (cardiolipin) in a 67:23.2:9.8 ratio (weight percent). BLMs were formed from a 5% lipid solution in n-decane by the Mueller [39] technique on a 0.85 mm orifice in a Teflon partition separating two compartments of 2 mL each. Peptides in the buffer were added to both compartments to reach final concentrations of 1 μM. The measurements were taken in 5 mM HEPES and 0.154 M NaCl at pH 7.4 and 25 °C. The current records were digitized at a 10 kHz frequency and analyzed using ClampFit (version 9.2; Axon Instruments Inc., Burlingame, CA, USA). Pore diameter (Dp) was calculated using the formula $D=2*\sqrt{\frac{g\;*\;L}{\pi \;\sigma }},$ where L is the thickness of a membrane (50 Å), g is the measured conductance, and σ is the conductance of an electrolyte (1.24 S/m).

## 4. Conclusions

The low cytotoxicity profile of capitellacin allowed the improvement of its selectivity through activity buildup. The corresponding effects were driven by the addition of an *N*-terminal arginine residue (CT2) or the removal of terminal residues (CT4). Nevertheless, the produced chimeric compounds did not express a significant increase in the selectivity. Crucially, observational changes in the kinetics of action on the bacterial membrane were achieved by modifying the β-turn region (CT7), but they were also accompanied by an increase in nonspecific cytotoxicity. The double amino acid substitution in the β-turn (R8Y, N9R) of capitellacin was believed to cause a change in the mechanism of its action. The hydrophilic nature of the β-hairpin region tends to prevent the transmembrane orientation of capitellacin in contrast to tachyplesin-1 which acts via the formation of multiple transmembrane pores. Unlike tachyplesin-1, capitellacin predominantly acts via the “carpet” or detergent-like mechanism of action. The peptide accumulates on the membrane surface, causing small fluctuations in conductivity and, when the threshold concentration is reached, leads to its complete destruction.

Capitellacin is an attractive scaffold for potential therapeutic application due to its biological properties. In addition to its ability to kill pathogens via the membranotropic mechanism of action, which ultimately minimizes the likelihood of developing resistance, it also exhibits anti-biofilm activity. Despite the fact that capitellacin acts at various stages of biofilm formation, further research is needed to develop a deeper understanding of its anti-biofilm mechanism of action.

## Figures and Tables

**Table 1:** Characteristics of the recombinant AMPs.

Peptide	Recombinant Peptide Final Yield, mg/L	RP-HPLC Retention Time, min ^1^	Hydrophobicity Index ^2^	Calculated [M+H]^+^ Monoisotopic Mass, Da ^3^	Measured Monoisotopic *m*/*z* Value ^4^
Cap *	6.1	38.5	−0.215	2379.16	2379.27
CT2	5.5	38.0	−0.419	2535.26	2535.28
CT3	5.1	38.3	−0.205	2322.13	2322.31
CT4	5.2	37.8	−0.388	2138.05	2138.54
CT5	5.1	33.5	−0.565	2167.08	2167.42
CT6	6.4	37.7	−0.188	2172.03	2172.32
CT7	4.6	40.8	0.041	2187.07	2187.43
Tach-1	7.2	37.5	−0.518	2264.10	2263.73

* Capitellacin. ^1^ Retention times were measured using semipreparative reversed-phase high-performance liquid chromatography (RP-HPLC) on a C18 column with a linear gradient from 5 to 80% (*v/v*) acetonitrile in water containing 0.1% trifluoroacetic acid (TFA) within 1 h. ^2^ Mean Kyte–Doolittle hydrophobicity index (GRAVY) was calculated using the Expasy ProtParam tool. The maximum and minimum values of this index are +4.5 and 4.5 for poly-Ile and poly-Arg sequences, respectively. ^3^ Molecular masses were calculated by considering the presence of four Cys residues forming two disulfide bonds. ^4^ Molecular masses were determined experimentally using MALDI-TOF mass spectrometry.

**Table 2:** Minimum inhibitory concentration (MIC) of peptides against Gram-positive and Gram-negative bacteria.

Bacteria	Minimum Inhibitory Concentration (µM)							
	Cap *	CT2	CT3	CT4	CT5	CT6	CT7	Tach-1
Gram-positive								
*Micrococcus luteus* B-1314	4	1	4	4	8	4	1	2
Staphylococcus aureus 209P	8	4	8	4	8	4	2	1
*Bacillus subtilis* B-886	16	8	32	16	32	16	4	2
Geometric mean (µM) **	8	3.17	10.08	6.35	12.7	6.35	2	1.59
Gram-negative								
*E. coli* MDR CI 1057	0.5	n.d.	n.d.	n.d.	n.d.	n.d.	n.d.	0.125
*E. coli* BW25113	0.5	n.d.	n.d.	n.d.	n.d.	n.d.	n.d.	0.25
*E. coli* ML-35p	0.5	0.25	0.5	0.25	0.5	0.25	0.5	0.125
*E. coli* ATTC 25922	0.5	0.25	0.5	0.5	0.5	0.25	0.25	0.25
*E. coli CI 3600*	0.5	0.25	0.5	0.25	0.5	0.25	0.25	0.06
*E. coli CI 214*	1	0.25	1	0.5	0.5	0.25	0.25	0.06
*E. cloacae* CI 4172	4	2	8	4	4	2	2	0.25
*A. baumanii* CI 2675	0.25	0.125	0.25	0.125	0.5	0.25	0.125	0.016
*P. aeruginosa* PAO1	2	1	2	1	4	2	1	0.5
*K. pneumonia* ATCC 700603	4	2	4	2	4	2	4	1
Geometric mean (µM) **	1	0.46	1.09	0.59	1.09	0.55	0.55	0.15

n.d., not determined. * Capitellacin. ** The geometric mean (GM) of the peptide MICs against test strains.

## Data Availability

Not applicable.

## Supplementary Materials

The following supporting information can be downloaded at: https://www.mdpi.com/article/10.3390/md20030167/s1, Figure S1: Reverse-phase high-performance liquid chromatography purification of peptides; Figure S2: Repurification of peptides was performed using the analytical column; Figure S3: Anti-biofilm activity of capitellacin and tachyplesin-1 against *A. baumanii* XDR CI 2675, *K. pneumonia* ATCC 700603 and *P. aeruginosa* XDR CI 1995.
