# Supplementary material for: Mechanism of Action and Therapeutic Potential of the β-Hairpin Antimicrobial Peptide Capitellacin from the Marine Polychaeta Capitella teleta
Source: Mar Drugs. 2022 Feb 25;20(3):167. doi: 10.3390/md20030167 (PMC8953592; doi:10.3390/md20030167)
Supplement: Supplementary file 1 [file marinedrugs-20-00167-s001.zip › marinedrugs-1601085-supplementary.pdf]

Supplementary material

Mechanism of action and therapeutic potential of the β-hairpin antimicrobial peptide capitellacin from the marine polychaeta *Capitella teleta*

Victoria N. Safronova[1], Pavel V. Panteleev[1], Stanislav V. Sukhanov[1], Ilia Y. Toropygin[2], Ilia A. Bolosov[1], Tatiana V. Ovchinnikova[1]*

[1] M.M. Shemyakin & Yu.A. Ovchinnikov Institute of Bioorganic Chemistry, the Russian Academy of Sciences, Miklukho-Maklaya str., 16/10, 117997 Moscow, Russia;

[2] V.N. Orekhovich Research Institute of Biomedical Chemistry, Pogodinskaya str., 10, 119121 Moscow

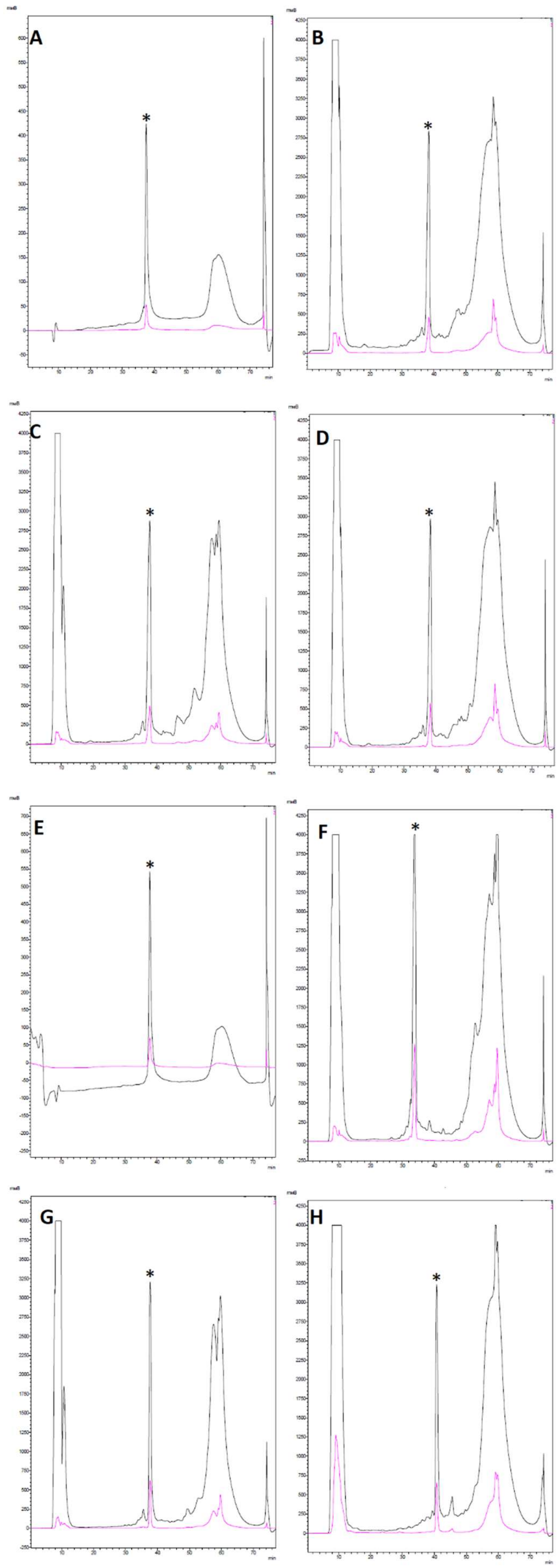

Figure S1. Reverse-phase high-performance liquid chromatography (RP-HPLC) purification of A) Tachyplesin-1; B) Capitellacin; C) CT2; D) CT3; E) CT4; F) CT5; G) CT6; H) CT7. The fraction of recombinant peptide is marked with a black asterisk.

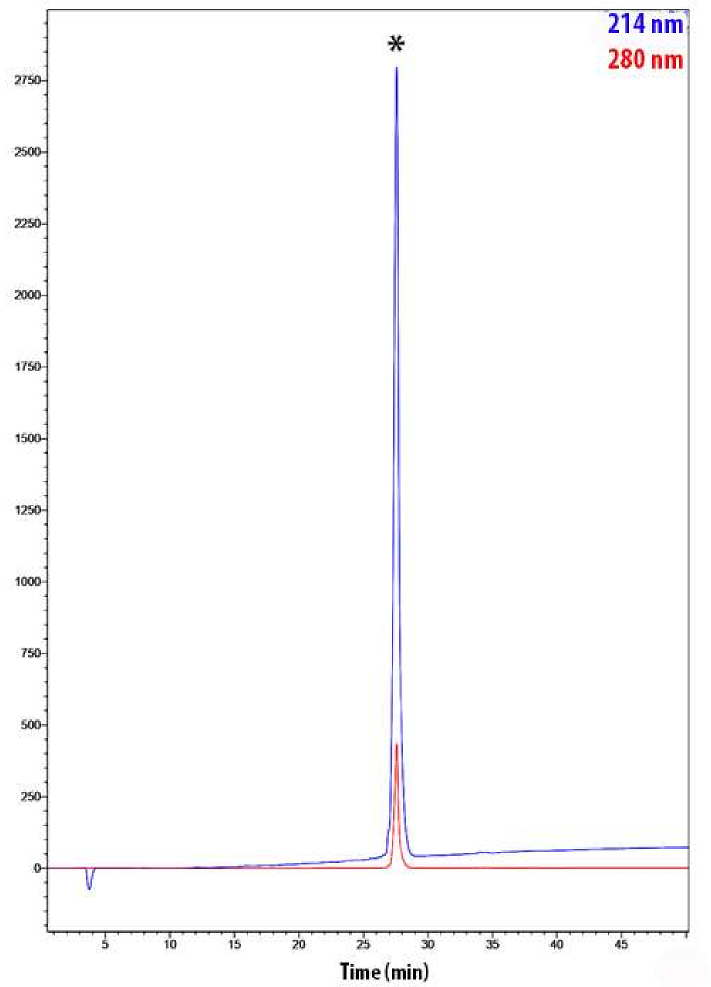

Figure S2. Repurification of peptides was performed using the analytical column (Symmetry 300 C18) at a flow rate of 1 mL/min in a linear gradient of solution B (80% acetonitrile, 0.1% TFA) in solution A (5% acetonitrile, 0.1% TFA): 0-100% for 50 min. (capitellacin is given as an example).

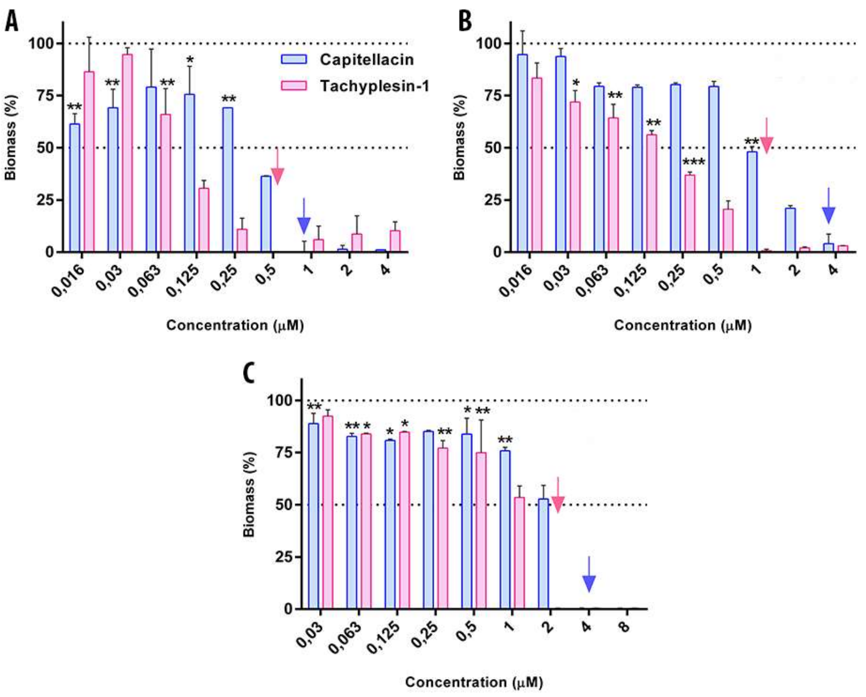

Figure S3. Anti-biofilm activity of capitellacin and tachyplesin-1 against A) *A. baumanii* XDR CI 2675; B) *K. pneumonia* ATCC 700603; C) *P. aeruginosa* XDR CI 1995. (CI, clinical isolate. MDR, multidrug resistant strain. XDR, extensively drug resistant strain). Up arrow is MIC of peptide. ± SD of at least two independent experiments, statistically significant differences vs. the control: $^{*}p \leq 0.05$, $^{**}p \leq 0.01$, $^{***}p \leq 0.001$.
